# Contribution to ecological environmental factors and the occurrence of haemosporidians in birds in Zhongar Alatau National Park, Kazakhstan

**DOI:** 10.1007/s00436-023-08097-7

**Published:** 2023-12-23

**Authors:** Martina Haas, Lenka Ploščicová

**Affiliations:** Institute of High Mountain Biology, Žilina University, Tatranská Javorina 7, 059 56 Tatranská Javorina, Slovakia

**Keywords:** Avian haemosporidian, *Haemoproteus*, *Leucocytozoon*, Passerine, Ecological conditions, Zhongar Alatau

## Abstract

In addition to the presence of a suitable host and vector, the prevalence of haemosporidians is influenced by several important factors, including the environmental conditions of the habitat, which depend on broader geographic characteristics. The aim of this study is to perform a preliminarily assessment of the distribution of blood parasites in birds from the mountainous area of Zhongar Alatau NP and to find potential new sites for research on their ecology in Kazakhstan. The results of this research constitute the first report on the occurrence of blood parasites from this area. A total of 58 birds, from the order Passeriformes and one individual from the order Caprimulgiformes, were examined during the study. The overall prevalence of infections caused by haemosporidian parasites (*Haemoproteus*, *Leucocytozoon*) was 18.6%. Neither the genus *Plasmodium* nor the presence of trypanosomes and microfilariae was detected in the birds examined. Three birds (5.1% prevalence) were infected with parasites of the genus *Haemoproteus*, in all eleven positive birds the analyses showed the presence of parasites of the genus *Leucocytozoon* (18.6% prevalence). The presence of parasites genus *Haemoproteus* was detected only in birds that were also infected with *Leucocytozoon* parasites. More infections with parasites of the genus *Leucocytozoon* are predicted due to the higher altitude and ecological factors at the capture sites, which are more favourable for the development of vectors of this genus. The species *Haemoproteus majoris* was detected in the host *Emberiza cioides* and species *Haemoproteus minutus* in host *Turdus merula.* Other species of this genus in the hosts *Cyanistes cyanus* and *Turdus atrogularis* were not determined. The species *Leucocytozoon fringilinarum* was detected in the hosts *Cyanistes cyanus* and *Parus major*, *Leucocytozoon dubreuili* was detected in *Turdus atrogularis* and *Turdus merula*. In the other host species *Aegithalos caudatus, Emberiza cioides *and* Periparus aterus*, it was not possible to dermine the species of the genus *Leucocytozoon*.

## Introduction

The study of haemosporidian parasites is a frequently discussed topic in current parasitological research, particularly in avian malaria research, which has undeniable advantages in answering ecological, behavioural and evolutionary questions (Valkiūnas 2005; Marzal et al. 2011). The largest group of haemosporidian consists of the avian haemosporidians, and more than 200 species of parasites from the genera *Plasmodium, Haemoproteus* and *Leucocytozoon* have been described based on their morphological features (Valkiūnas 2005; Levine 2018; Valkiūnas and Iezhova 2018, 2022).

Bird haemosporidian parasites have a worldwide distribution, and apart from Antarctica, have been found in all zoogeographic regions, in each of the landscape zones and in all bird clades. Their dispersal includes mountains up to 3000 m a.s.l., and some species are actively transported beyond the Arctic Circle (Valkiūnas 2005). These vector-borne pathogens pose negative effects on the fitness and survival of infected birds (Bensch et al. 2009), because this group of intracellular blood parasites may cause a dramatic reduction in metabolic efficiency (Chen et al. 2001). In geographic areas where host species have not evolved with haemosporidian parasites, primarily due to no or low abundance, birds are particularly sensitive to haemosporidian infections, often resulting in host mortality (Atkinson 2005; Grilo et al. 2016; Vanstreels et al. 2016; Inumaru et al. 2020).

Species identification based on the morphological parameters of parasites detected in blood smears is quite difficult, especially in wild birds, which show low parasitaemia at the time of capture (Oliveira et al. 2020). The introduction of molecular methods of diagnosis (e.g.Bensch et al. 2000, 2009; Sehgal 2015) has also helped to advance haemosporidian research. Despite the relatively high number of specific studies on host-parasite interactions (e.g.Yusupova et al. 2023; Caizergues et al. 2023; Attaran et al. 2021), parasite diversity (e.g.Oliveira et al. 2020; Ciloglu et al. 2020; Schumm et al. 2021), life cycle (e.g.Ilgūnas et al. 2019, 2022; Cepeda et al. 2019; Valkiūnas and Atkinson 2020) and ecology (e.g.Strehmann et al. 2023; Theodosopoulos et al. 2023), geographic distribution has not been studied equitably in all regions, and there are still areas where the occurrence of bird haemosporidian has not yet been documented.

Although the distribution of haemosporidian varies between regions, irregular studies within regions lead to an analysis of the distribution of avian haemosporidian by major zoogeographic regions (Valkiūnas 2005). According to Clark (2018), the most species-rich regions for haemosporidian diversity are the Eurasian, Amazonian, African and North American zoogeographical regions. However, these conclusions are mainly a result of the number of bird species studied, and the higher number of studies conducted in European and North American countries than on ther continents (Clark et al. 2014).

The distribution of haemosporidians in host bird species in Central Asia is poorly studied and remains unknown in many areas, but this distribution is also largely unknown, also in representatives of some orders and families of birds. Latest information on the distribution and abundance of parasites, wild hosts and vectors are lacking from many regions of Kazakhstan. Studies from the second half of the twentieth century provide information on 20.8% prevalence in wild and 8% in domestic bird species (Yakunin 1976; Yakunin and Zhazyltaev 1977). However, they do not present a complete picture of the species diversity of blood parasites in birds, their degree of infection, restriction to a particular host species, or localization and distribution within regions of the country. Kairullaev (1986, 2010) reports the detection of 14 species of *Plasmodium*, 45 species of *Haemoproteus* and 23 species of *Leucocytozoon* based on his own investigations and data from the available literature. He also reported that *Plasmodium* was found in 102 (36.6%), *Haemoproteus* in 163 (58.6%) and *Leucocytozoon* in 125 (44.9%) out of a total of 278 bird species studied. More recent studies from Kazakhstan have focused on the occurrence of haemosporidian in some host species, such as bird of prey (Leppert et al. 2004; Sehgal et al. 2006) or in breeding songbirds (Valkiūnas et al. 2006; Zehtindjiev et al. 2009).

As there are no recent data on the species composition of haemosporidian in wild birds of mountainous area in Kazakhstan, this study will contribute to the knowledge base on the distribution of these parasites. The aim of this study, which we conducted during short-term monitoring of birds in the Zhongar Alatau region, is to begin to assess the distribution of blood parasites in birds in this area and to find potential new sites for research on their ecology in Kazakhstan.

## Material and methods

### Study area and sampling

The birds were captured from 14 to 20 September 2022 in the mountainous areas of the Zhetysu region, in Zhongar Alatau National Park, Kazakhstan. This area of high biodiversity belongs to the Tian Shan steppe and meadow mountain ecoregion—WWF ID PA 1019 (Carpenter 2000); it extends from steppes through alpine meadows to glaciers and forms a transition zone between boreal, steppe and desert areas. Although the forests are predominantly comprised of pine and spruce, the park contains significant stands of wild fruit trees (900–1800 m a.s.l.), particularly the Siever’s apple (*Malus Sieversii*), which is the ancestor of all cultivated apple varieties in the world (Dzhangaliev 2003; IUCN 2015; Bakhtaulova et al. 2015).

Research was conducted in two locations: Osinovaya (45.40526 N; 80.40581 E) located in an apple forest at an altitude of 1207 m a.s.l. and Bashkan (45.2653758 N; 80.1525908 E; 1492 m ass.l.) representing the forest to subalpine zone.

Birds were captured in ornithological nets, and blood was collected from captured birds (*n* = 59) by puncturing the brachial vein. A drop of blood was transferred to a glass slide and a blood smear was created. Remaining blood was deposited on a cellulose swab (Pur-Zellin®, Hartmann A.G., Germany). The sample (blood stain) was air-dried and stored sterilely for further analysis. After examination, the birds were released at the site of capture.

### Laboratory examination

DNA for bird blood parasites was isolated from each dried blood stain. Approximately three-by-three mm of each blood stain was cut using sterile scissors. Commercially available DNeasy Blood & Tissue Kits (QIAGEN, Germany) were used for DNA extraction according to the manufacturer’s protocol. DNA samples were analysed for the content of *Leucotyzoon* sp., *Haemoproteus* sp. and *Plasmodium* sp. with the use of PCR. This was done using primers and nested PCR using the protocol developed by Hellgren et al. (2004). The initial primers Haem NFI (5′-CATATATTAAGAGAAITATGGAG-3′) and Haem NR3 (5′-ATAGAAAGATAAGAAATACCATTC-3′) were used to amplify mitochondrial DNA of haemosporidian parasites (cytochrome b gene, 617 bp fragment). The first PCR was performed according to the mentioned protocol (Hellgren et al. 2004), with the minor changes as follows: 5 × master mix FIREPoL, 0.4 pM forward primer, 0.4 pm reverse primer, 3 μL DNA template and distilled water to a 20 μL volume. PCR conditions were as follows: initial activation at 95 °C for 5 min; 30 cycles of denaturation at 95 °C for 1 min; annealing at 58 °C for 1 min; elongation at 72 °C for 1 min; and final elongation at 72 °C for 5 min. The product of the first PCR was taken (2 μl) as the template for the second PCR, 2 μl for *Leucocytozoon* sp. (HaemFL–HaemR3L) and 2 μl for *Haemoproteus* sp. and *Plasmodium* sp. (HaemF–HaemR2). These PCRs were performed separately in 20 μl volumes with the same reagent ratios as in the initial PCR reactions. The thermal profile of the PCRs was identical to the initial PCRs but was carried out over 35 cycles. Amplified PCR products were visualized using 2% agarose gel electrophoresis for 40 min at 80 V in1 × Tris–borate-EDTA buffer.

The expected-sized PCR products were sent to Macrogen (The Netherlands; http://www.macrogen.com) for purification and sequencing of both DNA strands. The sequences were edited in MEGA6 software (Tamura et al. 2013), and for identification of each of them was used Nucleotide Blast on NCBI. Sequences having double peaks, which indicate co-infections, were designated as DP, and sequences of poor quality were designated as unusable sequences (US).

Blood smears were stained according to the Pappenheim method (Doubek et al. 2003). Microscopic examination of the blood was performed using a Leica DM6000B light microscope (Leica Microsystems, GmbH). In each smear, more than one hundred fields and 50,000 erythrocytes were observed at 1000 × magnification.

Microscopic screening using the LAS attachment and software (Leica Application Suite; ver. 4.5.0; Leica Microsystems CMS GmbH) was used to determine the species of each parasite, and species identification based on morphological features of fully developed gametocytes was performed according to the Key for Identification of Hemosporidia (Valkiūnas 2005; Valkiūnas and Iezhova 2022).

## Results

**Fig. 1:**
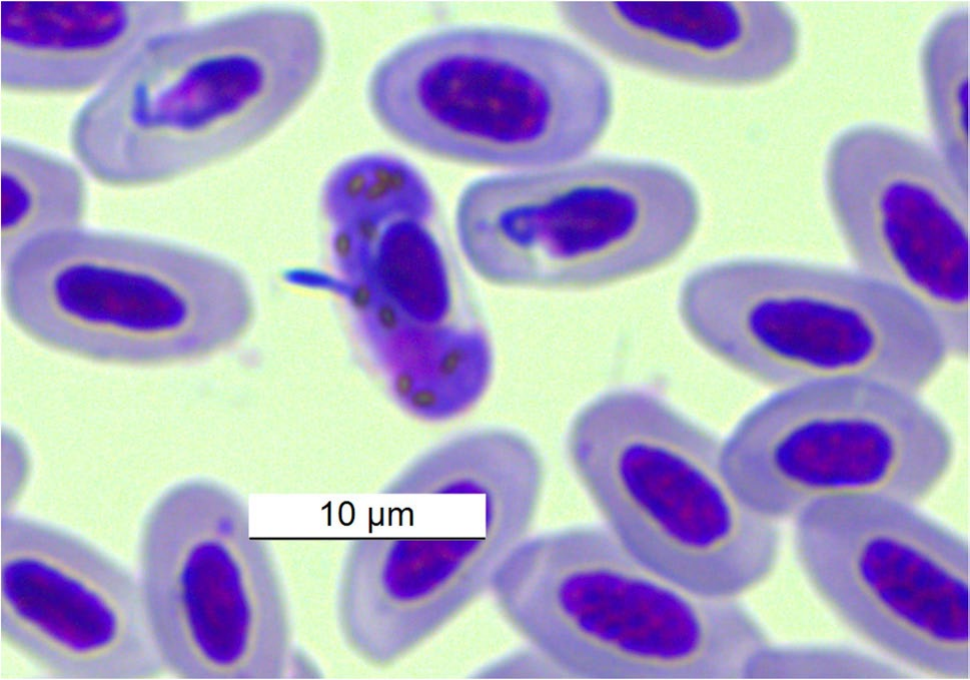
*Haemoproteus majoris *from host species *E. cioides* captured at Bashkan, Kazakhstan

**Fig. 2:**
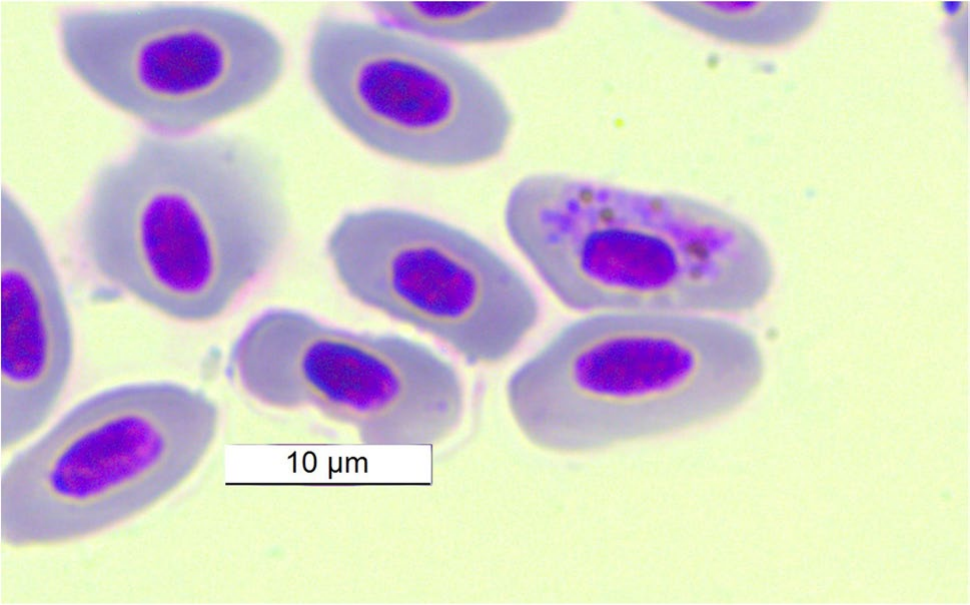
*Haemoproteus* *minutus* from host species *T. merula* captured at Bashkan, Kazakhstan

**Fig. 3:**
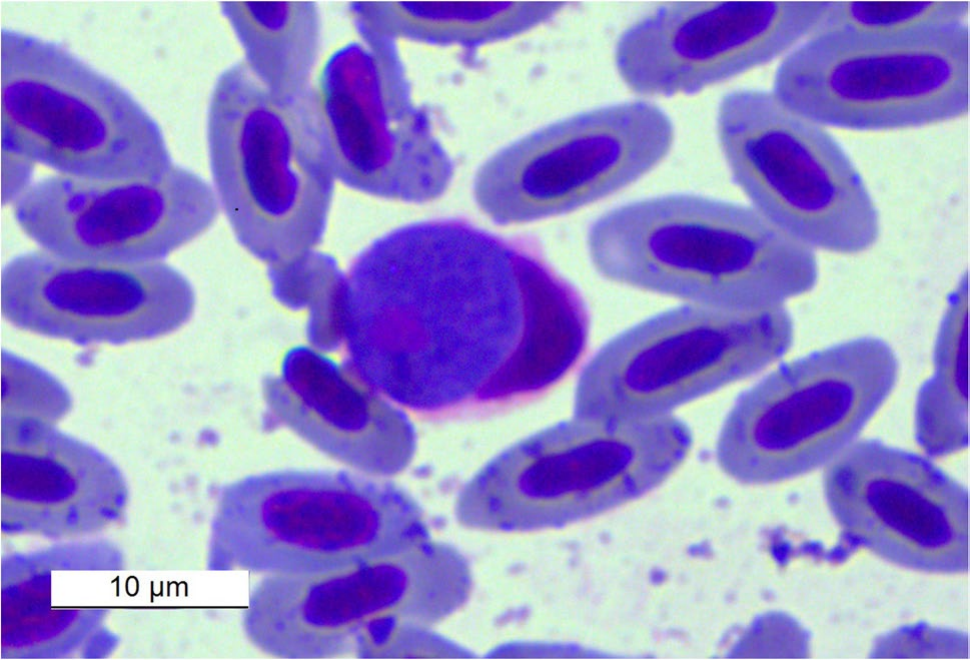
*Leucocytozoon fringillinarum* from host species * C. cyanus *captured at Osinovaya, Kazakhstan

**Fig. 4:**
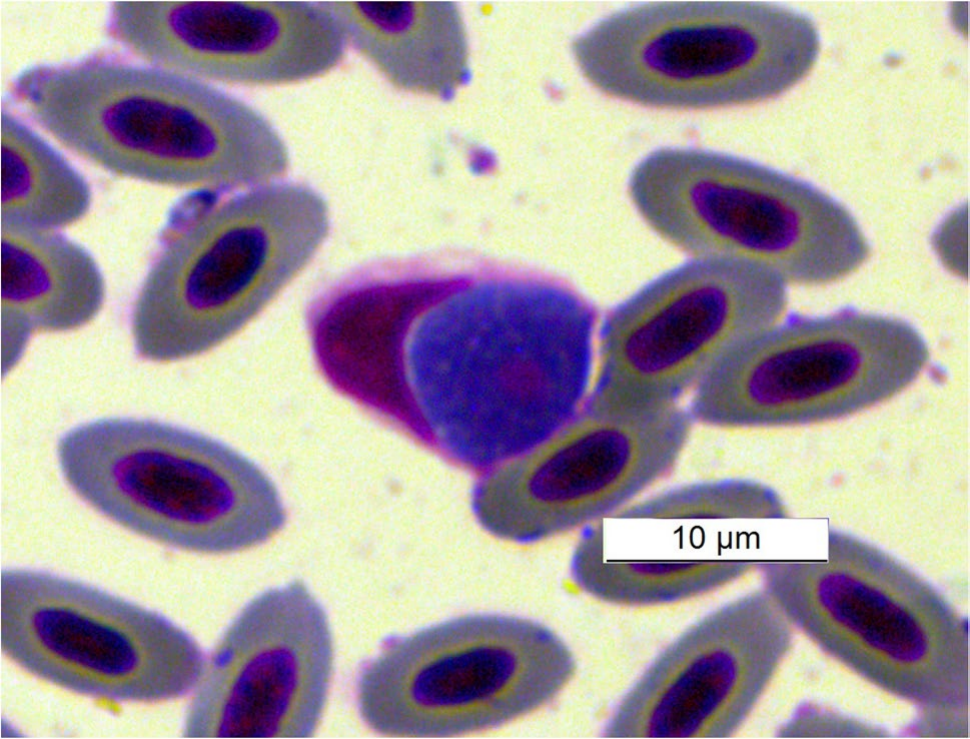
*Leucocytozoon* *fringillinarum* from host species * P. major* captured at Osinovaya, Kazakhstan

**Fig. 5:**
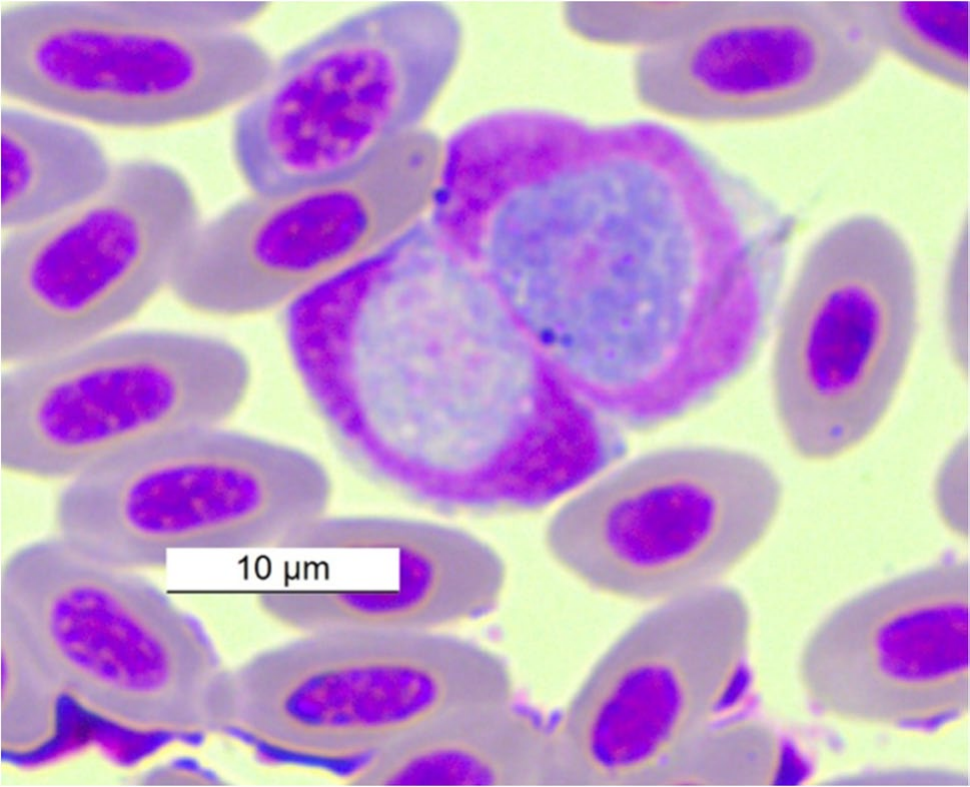
*Leucocytozoon dubreuli* from host species *T. merula* captured at Osinovaya, Kazakhstan

PCR analyses and sequencing of positive samples confirmed the presence of parasite lineages (Table 1) for the genera *Haemoproteus* and *Leucocytozoon*. The overall prevalence of avian haemosporidians was 18.6% (11 infected out of 59 examined). Parasites of the genus *Haemoproteus* infected 3 birds (5.1% prevalence) of the host species *Emberiza cioides* (Brandt, 1843), *Turdus atrogularis* (Jarocki, 1819) and *Turdus merula* (Linnaeus, 1758). The occurrence of parasites of the genus *Haemoproteus* were detected only as mixed infections in birds infected with parasites of the genus *Leucocytozoon*. Microscopic examination determined the species of *Haemoproteus* *majoris* (Laveran, 1902) in *E. cioides* (Fig. 1) and *Haemoproteus minutus *(Valkiūnas and Iezhova, 1992) in *T. merula *(Fig. 2). Parasites of the genus *Leucocytozoon* were found in all 11 individuals (18.6%) infected. The host species *E. cioides,* *Aegithalos caudatus *(Linnaeus, 1758), *Cyanistes cyanus* (Pallas, 1770), *Parus major* (Linnaeus 1758); *Periparus ater* (Linnaeus, 1758), *Turdus merula *(Linnaeus, 1758) and *T. atrogularis. *By microscopic examination, it was possible to determine the species *Leucocytozoon fringillinarum* (Woodcock, 1910) in hosts of* C. cyanus* (Fig. 3) and* P. major* (Fig. 4) and* Leucocytozoon dubreuili* (Matis and Léger, 1911) in hosts of* T. atrogularis* and *T. merula* (Fig. 5). In the hosts *P. major* and *C. cyanus* (both infected birds), the sequences were of low quality (unusable sequences); therefore, no lineas are identified. Co-infection with at least 2 species of *Leucocytozoon* parasites (double peak) was detected by sequencing in the host *T. merula*, one species could be microscopically determined as *L. dubreuili*.

**Table 1:** Host bird species, prevalence and identified haemosporidian species and haemosporidian lineages according to GenBank.. *DP* double peaks (co-infections), *US* unusable sequences

Species	Site	No. of exam. birds	No. of infected birds	Prevalence	Determination of haemopsporidian parasite	Haemosporidian lineage
Order: Caprimulgiformes						
Family: Caprimulgidae						
*Caprimulgus europeus*	Osinovaya	1	0			
Order: Passeriformes						
Family: Aegithalidae						
*Aegithalos caudatus*	Osinovaya	7	2	28.6%	*Leucocytozoon* sp.*	LC701766.1 KJ488788.1 GU391354.1
Family: Emberizidae						
*Emberiza cioides*	Bashkan	1	1	100%	*Haemoproteus majoris* *Leucocytozoon sp.*	MK652255.1 ON138438.1 KJ488788.1 MN459538.1
Family: Fringilidae						
*Fringilla coelebs*	Osinovaya	1	0			
*Chloris chloris*	Osinovaya	1	0			
Family: Paridae						
*Cyanistes cyanus*	Osinovaya	3	2	66.7%	*Haemoproteus *sp.* *Leucocytozoon* sp. *Leucocytozoon fringillinarum*	US LC440380.1 MG649340.1
*Parus major*	Osinovaya	23	1	4.3%	*Leucocytozoon fringillinarum*	LC440380.1
*Periparus ater*	Osinovaya	7	2	28.6%	*Leucocytozoon* sp.*	LC440380.1**
Family: Phylloscopidae						
*Phylloscopus collybita tristis*	Osinovaya	1	0			
*Phylloscopus humei*	Osinovaya	3	0			
Family: Sylviidae						
*Curruca althaea*	Osinovaya	2	0			
Family: Turdidae						
*Turdus atrogularis*	Osinovaya	7	1		*Haemoproteus* sp. *Leucocytozoon dubreuili*	MN104973.1 US
*Turdus merula*	Osinovaya Bashkan	1 1	1 1	100%	*Leucocytozoon dubreuili* *Leucocytozoon dubreuili* *Haemoproteus minutus*	KJ488788.1 DP KY653763.1, KJ488583.1, KF192999.1, KF192995.1 DQ630013.1
Total	59	11	18.6%			

*Inference from PCR diagnostics

**Same lineage for both infected birds

Microscopic examination of blood smears did not reveal the presence of trypanosomes and microfilariae. Infection with parasites of genus *Plasmodium* was not detected by microscopic examination or molecular methods.

## Discussion

### Host species and species distribution of haemosporidian

This study is the first report on the occurrence of blood parasites from the territory of Zhongar Alatau NP, located in the eastern part of Kazakhstan. Kairullaev (2010) reports in his research that for 2371 birds examined (87 species) from the main migratory routes of birds in Kazakhstan, the overall prevalence of blood parasites was 19.4 ± 0.8%. In addition to other blood parasites present in the peripheral blood of birds,*Plasmodium* (1.6%), *Haemoproteus* (16.6%) and *Leucocytozoon*(1.1%) were found in 25, 57 and 19 species of birds, respectively. Despite the significantly lower number of birds examined in our study, it is surprising that the overall prevalence (18.6%) is close to the value reported by Kairullaev (2010). The higher prevalence of genus *Leucocytozoon* in our study can be explained by ecological factors at the capture site, as detailed below.

Yakunin and Zhazyltaev (1977) confirmed *P. major, T. atrogularis* and *T. merula* as host species from the territory of Kazakhstan, while *P. ater* and *E. cioides* were not found to have haemosporidian in the examined samples. Similarly, the presence of haemosporidian in *E. cioides* was not observed in the studies of Sodhi et al. (1999), but Inumaru et al. (2022) confirmed the presence of *Haemoproteus* sp. in one of three individuals examined. In our samples, based on morphological characteristics, we detected *Haemoproteus majoris* in this species, and therefore, we propose to include *E. cioides* among the host species of* H.* *majoris*. *A.* *caudatus* is also one of the bird species in which the presence of haemosporidian have not yet been confirmed (Valkiūnas 2005; Inumaru et al. 2022). In the host species *C. cyanus*, we detected positive samples on *Haemoproteus* by molecular analyses (lineages could not be determined due to poor sequence quality) in two birds examined. Sequencing revealed positive *Leucocytozoon* lineages in both positive individuals as well. In one individual (lineage MG649340.1), microscopic determination of *L. fringilinarum* was possible. Species determination by microscopy was not possible as no adult forms of gametocytes were found in the blood smears. We also confirmed the same species *L. fringillinarum* in the host *P. major*, which is an atypical finding for this species, as it tends to be infected earlier by *L. majoris* (Valkiūnas 2005). Although *P. major* is one of the hosts in which detection of haemosporidian is relatively common, prevalences vary among years, seasons and regions (e.g.Allander and Bennett 1994; Norte et al. 2009; Schumm et al. 2019). *Leucocytozoon dubreuili* was found in host species *T. atrogularis* and *T. merula,* based on morphological characters. This species is common in *Turdus* sp. (Valkiūnas 2005).

Even molecular analyses did not confirm parasites of the genus *Plasmodium* in the birds studied. The low prevalence of this genus in birds in Kazakhstan is also reported by Kairullaev (2010). The occurrence of the genus *Plasmodium* is less frequent in the Holarctic zoographic region compared to the genera *Haemoproteus* and *Leucocytozoon* (Valkiūnas 2005).

### Ecological factors of the presence of the haemosporidian parasites

The overall prevalence of parasites in a bird community is influenced by host composition, but as some studies suggest, geographic conditions (Scordato and Kardish 2014) or habitat characteristics may also be important in predicting the prevalence of blood parasites. However, this tends to be overlooked by many studies (Sehgal 2015). Local environmental factors provide the conditions for development of the parasitic system of avian haemosporidian, including susceptible hosts and vectors. Environmental conditions, including temperature and the presence of water resources, are particularly important for the development of vectors.

We detected two genera of haemosporidian *Haemoproteus* and *Leucocytozoon* in the examined. These genera, according to Valkiūnas (2005), are more widely represented in the Holarctic zone. Leucocytozoonoses are more widespread within this region in mid to high-latitude areas, due to the ability of the parasites to complete development in vectors at relatively low temperatures (Valkiūnas 2005). Higher prevalence of the genus *Leucocytozoon* is confirmed in mountainous regions or at higher altitudes (Haas et al. 2012; Imura et al. 2012; Rooyen et al. 2013; Lotta et al. 2015), in alpine regions of North America (Murdock 2009; Oakgrove et al. 2014) and in northern regions of Europe (Scheuerlein and Ricklefs 2004). The capture of birds took place at altitudes of 1200 and 1490 m a.s.l., and the higher incidence of leucocytozoonoses can also be explained by this factor. According to Illera et al. (2017), this parasite’s prevalence and abundance showed a positive association with temperature and a negative association with rainfall. Their results suggest that infections by parasites of the genus *Leucocytozoon* occur more often in warm and dry forest, consistent with our results. The continental climate in Kazakhstan is influence by it’s remoteness from the ocean and high radiation, and it can also be characterized as a cold, semi-arid climate (Kottek et al. 2006), though it varies significantly with elevation in the mountains. The climate in Khazakhstan is characterized by long and severe winters, short hot summers, a high quantity of clear days, higher aridity and variable temperatures. The typical mountain climate is characterized by significant diurnal and seasonal temperature variations, uneven distribution of rainfall throughout the year and moderate humidity. Sunny and dry weather prevailed during the trapping days of our research.

The dynamics of avian haemosporidian occurrence depend on selective pressures that vary in space and time (Lynton-Jenkins et al. 2020). Haemosporidian from areas with well-defined seasonal changes are characterized by increased occurrence during periods that overlap with the highest vector frequency (i.e. in the temperate zone during spring). *Haemoproteus* parasites have a secondary peak of increased parasitemia specifically during autumn (Krone et al. 2001), although these peaks do not occur simultaneously in different territories (Valkiūnas 1987a). However, the spring invasion of haemosporidian is significantly higher than the autumn invasion (Valkiūnas 1987b). Despite an increase infection with parazites of the genus *Haemoproteus* during autumn, we only confirmed three cases in our samples.

The quality of the host habitat is characterized by microclimate, latitude and/or altitude as well as landscape type. Seasonal changes in temperate climatic conditions also integrate changes in environmental factors such as rainfall, humidity and ambient temperature. The development and activity of the vectors of avian haemosporidians are directly dependent on these environmental factors. The seasonal occurrence of blood parasites is dependent on the abundance and activity of vectors, which are conditioned by environmental factors in changing seasons. These environmental factors directly influence the presence, development, abundance, distribution and activity of vectors, which is reflected in the prevalence of blood parasites (Shutler et al. 1999; Krone et al. 2001; Hauptmanová et al. 2002; Wilson et al. 2001; Shocket et al. 2021). Vector dynamics may also vary between years when the species composition of vectors varies depending on suitable climatic conditions (Svobodová and Votýpka 1998). Likewise, behavioural and feeding strategies of vectors vary in different climatic zones (Valkiūnas 1987b). Kairullaev and Yakunin (1982) found higher prevalence of blood parasites in the foothills of the western Tien Shan (Shakpak Ornithological Station), which can also be explained by more favourable climatic conditions for vector development.

Several studies have demonstrated that the composition of black flies is controlled by various environmental variables that are related to anthropogenic pressures on river landscapes (e.g. loss of riparian vegetation or frequent disturbances (Ya’cob et al. 2016a, b)). Furthermore, Simuliids, vectors of *Leucocytozoons*, are known to react to physical and chemical degradation, including acidification and organic pollution (Glötzel 1973; Seitz 1992), and can therefore serve as excellent indicators of water quality (Lautenschläger and Kiel 2005). It can be assumed that streams and rivers in Zhongar Alatau are less acidified and polluted, so they provide a more desirable environment for black flies as vectors of haemosporidians.

Parasites of genus *Haemoproteus* are transmitted by louse flies (Hippoboscidae) and biting midges (Ceratopogonidae) (Donovan et al. 2008; Wernery and Kaaden 2000; Masello et al. 2018). It is assumed that larvae and pupae of culicoid develop in wet and humid habitats of all types; therefore, the climate of Kazakhstan, characterized by low precipitation, is not suitable for them (Werner et al. 2020). Nevertheless, in longitudinal studies (Yakunin and Zhazyltaev 1977; Kairullaev 2010), they reach the highest prevalence among blood parasites. The location of these studies, which were conducted near lakes and rivers, may also contribute to these results. However, *Icosta minor* Bigot, 1858 of the family Ceratopogonidae was recorded in large numbers in Kazakhstan between 1967 and 1972 (37 specimens, 34 from house and Spanish sparrows alone (Doszhanov 1975)), though they were not tested for the presence of haemoparasites (Jentzsch et al. 2021).

Among ecological conditions, distance from a water source is also a positive predictor for vector evolution (Mendenhall et al. 2013; Krama et al. 2015). Both study sites had mountain rivers and local streams or waterlogged areas, creating optimal conditions for vector development.

Environmental conditions in Zhongar-Alatau NP create a suitable environment for the evolution of host-parasite complexes within avian haemosporidian. The avifauna of the national park is represented by 238 species, of which 130 species make up the breeding bird complex. The area thus provides unique opportunities to study the ecological factors of haemosporidian distribution within intracontinental climatic conditions.

## Data Availability

All data generated or analysed during this study are included in this published article.
